# Rapid Mass Spectrometric Analysis of a Novel Fucoidan, Extracted from the Brown Alga *Coccophora langsdorfii*

**DOI:** 10.1155/2014/972450

**Published:** 2014-01-21

**Authors:** Stanislav D. Anastyuk, Tatyana I. Imbs, Pavel S. Dmitrenok, Tatyana N. Zvyagintseva

**Affiliations:** G.B. Elyakov Pacific Institute of Bioorganic Chemistry, Far Eastern Branch, Russian Academy of Sciences, 100 Let Vladivostoku Prosp., 159, 690022, Vladivostok, Russia

## Abstract

The novel highly sulfated (35%) fucoidan fraction **Cf2** , which contained, along with fucose, galactose and traces of xylose and uronic acids was purified from the brown alga *Coccophora langsdorfii*. Its structural features were predominantly determined (in comparison with fragments of known structure) by a rapid mass spectrometric investigation of the low-molecular-weight fragments, obtained by “mild” (5 mg/mL) and “exhaustive” (maximal concentration) autohydrolysis. Tandem matrix-assisted laser desorption/ionization mass spectra (MALDI-TOF/TOFMS) of fucooligosaccharides with even degree of polymerization (DP), obtained by “mild” autohydrolysis, were the same as that observed for fucoidan from *Fucus evanescens*, which have a backbone of alternating (1 → 3)- and (1 → 4) linked sulfated at C-2 and sometimes at C-4 of 3-linked **α**-L-Fuc*p* residues. Fragmentation patterns of oligosaccharides with odd DP indicated sulfation at C-2 and at C-4 of (1 → 3) linked **α**-L-Fuc*p* residues on the reducing terminus. Minor sulfation at C-3 was also suggested. The “exhaustive” autohydrolysis allowed us to observe the “mixed” oligosaccharides, built up of fucose/xylose and fucose/galactose. Xylose residues were found to occupy both the reducing and nonreducing termini of FucXyl disaccharides. Nonreducing galactose residues as part of GalFuc disaccharides were found to be linked, possibly, by 2-type of linkage to fucose residues and were found to be sulfated, most likely, at position C-2.

## 1. Introduction

The quantity of scientific papers, concerning various biological activities [1–4] of fucoidans, sulfated polysaccharides from brown algae, is constantly growing. However, one cannot notice the same about the works regarding the structural features of these unique carbohydrate polymers. Fucoidans are known [5] to be mainly built up of sulfated *α*-L-fucopyranose (*α*-L-Fuc*p*) residues. Other monosaccharides, like D-galactose, can occupy significant percentage in the monosaccharide composition in galactofucans or reside in minor ones, like xylose [6–11]. In order to unambiguously establish the structure of such complex polysaccharides by powerful spectroscopic techniques, like ^13^C NMR, time-consuming multiple-step chemical modification procedures, coupled with purification steps, are required. These methods are offering low yields and thus they are requiring a large number of samples. Recently [12] it was found that modern methods of mass spectrometry, offering high sensitivity, accuracy, and speed, enabled the analysis of low-molecular-weight (LMW) oligosaccharides, left by solvolytic desulfation reaction as a side product. It was found that minor constituents of fucansulfate (Xyl, Gal, and GlcA residues) from the brown alga *Fucus evanescens* are incorporated into the backbone, probably, as branching points [12]. ^13^C NMR technique that was employed for the analysis of the same fucoidan [13] was unable to observe these fragments, just because they were rejected at purification step or the method was not sensitive enough. Hence, using mass spectrometry as a complementary tool to the spectroscopic techniques and methods of carbohydrate chemistry is essential for the elucidation of the exact structure of fucoidans. However, in some cases using known mechanisms of mass spectrometric fragmentation, it is possible to elucidate some structural features of fucoidans by mass spectrometry only. In this way glycosaminoglycans and carrageenans were successfully investigated by the analysis of their fragments, obtained either by enzymes or by acid hydrolysis [14–17]. Unfortunately, fucoidanases are still to come, they are not widely available [18]. Despite of that, a polymer chain must be “softly” depolymerized to save“intact” positions of labile sulfate groups and other substitutes. An autohydrolysis (“autohydrolysis” is used here to denote acidic polysaccharide hydrolysis under very mild conditions using the –SO_3_H groups of the compound as the source of acid) procedure was developed as an alternative to the mild acid hydrolysis decomposition technique. It was found that it is a reproducible method for the preparation of multisulfated oligosaccharides, well reflecting the structure of the source polysaccharide. In this way, fragments of fucoidans from brown algae *Saccharina cichorioides *[19], *F. evanescens* [1], and *Costaria costata* [20] were successfully analyzed by tandem ESI- and MALDI-TOFMS. Structural features of oligosaccharides matched with that observed previously for corresponding fucoidans using independent methods [13, 21].

Herein we report mass spectrometric elucidation of the structural properties of oligosaccharides, obtained by “soft” and “exhaustive” autohydrolysis from a novel fucoidan, purified from the brown seaweed *Coccophora langsdorfii*.

## 2. Experimental

### 2.1. Materials

Brown alga *Coccophora langsdorfii* was collected from the Troitsa Bay (Sea of Japan) at the Sea Experimental Station of Pacific Institute of Bioorganic Chemistry (PIBOC) (http://goo.gl/maps/UMRce), Far-East Branch, Russian Academy of Sciences (Primorsky region of Russia) in July of 2008. The algae species were identified by Dr. A. Skriptsova (A. V. Zhirmunsky Institute of Marine Biology FEB RAS, Russia).

A MALDI-TOFMS matrix, 2,5-dihydroxybenzoic acid (DHB), was purchased from Sigma (USA). All experiments were performed using ultrapure water, produced with Direct-Q 3 equipment (Millipore, USA).

### 2.2. Instruments

MALDI-TOFMS spectra were recorded with an Ultra Flex III MALDI-TOF/TOF mass spectrometer (Bruker, Germany) with a nitrogen laser (SmartBeam, 355 nm), reflector, and potential LIFT tandem modes of operation.

Infrared spectra were recorded with a Vektor-22 (Bruker, Germany) FT-IR spectrometer.

Monosaccharide composition was determined on a Biotronik IC-5000 carbohydrate analyzer (Wissenschaftliche Geräte, Germany) using a Shim-pack ISA-07/S2504 (0.4 × 25 cm) column eluted with a potassium borate buffer at an elution rate of 0.6 mL/min. Detection was carried out by bicinchoninate method and integration on a Shimadzu C-R2 AX system. Monosaccharides (rhamnose, ribose, mannose, fucose, galactose, xylose, and glucose) were used as standards for HPLC.

### 2.3. General Methods

The content of carbohydrates was determined using the phenol-sulfuric method [22]. The content of sulfates in the polysaccharides was determined by a turbidimetric method [23].

#### 2.3.1. Extraction of Polysaccharides

The fresh samples of *C. langsdorfii* have been washed with deionized water in order to remove salt, epiphytes, and sand attached to the surfaces of the samples and dried between sheets of paper. Then, the algal biomass (1065 g) was treated at 40°C with ethanol (96% (v/v)) for 24 h to remove colored matter, washed with acetone, filtered, and vacuum dried to yield 230 g of defatted biomass. The milled defatted alga biomass (60 g) was extracted at 20–22°C with 0.1 M HCl (ratio 1 : 20) for 3 h. The extracts were combined, neutralized, dialyzed, and lyophilized to give crude polysaccharide fraction **Cf0** with yield 1.17 g (2% of dry defatted biomass). The monosaccharide composition of **Cf0** (mol%) is Fuc, 45.0, Xyl, 6.0, Man, 16.2%, Gal 18.0, Glc, 8.8, Uronic acids, and 6.0. The sulfate content of **Cf0 **is 18.2% from the fraction weight.

#### 2.3.2. Anion Exchange Chromatography

A solution of polysaccharides **Cf0** in 0.04 N HCl (1.0 g in 20 mL) was applied onto a DEAE-cellulose column (Cl^−^ form, 3.0 × 14 cm) equilibrated with 0.04 N HCl. Sulfated polysaccharides were successively eluted using NaCl-water solution liner gradient of 0 → 2 M. The fractions were dialyzed by ultrafiltration and lyophilized to get the polysaccharide preparations **Cf1** (43.5 mg) and **Cf2** (650 mg). The monosaccharide composition of the **Cf1** fraction (mol%) is Fuc, 45.8, Xyl, 8.0, Man, 16.2, Glc, 7.0, and Gal 23.0. The sulfate content of the **Cf1** fraction was not determined due to the low yield. The monosaccharide composition of the **Cf2** fraction (mol%) is Fuc, 86.0, Xyl, 3.0, Gal 6.9, and uronic acids 4.1. The sulfate content of the **Cf2** fraction was 35% from the fraction weight.

#### 2.3.3. Depolymerization of Fucoidan by Autohydrolysis

Autohydrolysis procedure [19] was used to obtain oligosaccharides suitable for MS analyses: an aliquot of 10 mL of fucoidan **Cf2** (5 mg/mL in water) was changed to the H^+^ form using a minicolumn of cation exchange (Timberlite CG-120, 200–400 mesh, Serva, Germany) and left for 72 h at 37°C. The mixture was then neutralized with 5% NH_4_OH solution in water and lyophilized to obtain **Cf2-AH** preparation. An exhaustive autohydrolysis was performed to obtain partially desulfated oligosaccharides: an aliquot of 10 mL of fucoidan **Cf2** (10 mg/mL in water) was changed to the H^+^ form as described above and lyophilized. Then, the sample was left at room temperature for 2 weeks (humidity ~80%) to obtain **Cf2-exAH** preparation.

#### 2.3.4. Derivatization of Oligosaccharides

NaBD_4_ reagent (Sigma, USA) of 50 *μ*L, 0.05 M NaBD_4_ in 0.01 M NaOH, was added to the lyophilized oligosaccharides (~50 *μ*g), and the reduction was carried out at 4°C overnight as described [24]. The solution was then neutralized with 0.1 mL AcOH and evaporated. Boric acid was removed by repeated coevaporation with MeOH.

#### 2.3.5. Mass Spectrometric Analysis

Instrument settings are as follows: accelerating voltage, 25 kV; laser power, 30 *μ*J; pulsewidth, 6 ns; number of shots, 100; laser shot rate, 66 Hz. Sample preparation is as follows: a mixture containing 1 *μ*L of sample (0.1 mg/mL) and 1 *μ*L of 0.5 M DHB matrix solution in MeOH was introduced onto the sample plate and air dried.

## 3. Results and Discussion

The present work is devoted to the preliminary characterization of a fucoidan from *C. langsdorfii* by the means of mass spectrometry. The brown alga *C. langsdorfii* (Order Fucales, Family Sargassaceae) is a perennial plant. It grows most actively in spring. Its biomass reaches maximum in June and January [25]. This alga is distributed in the Sea of Japan and in northern part of the East China Sea and can be found along the coasts of the Russian Far East [26, 27], Japan [25], and Korea [28]. The chemical composition of this alga is poorly studied. The polysaccharide composition of the *C. langsdorfii *is also studied at preliminary level [29].

### 3.1. Purification of Water-Soluble Polysaccharide Fractions and Preliminary Analysis

The crude polysaccharide preparation **Cf0**, extracted by cold extraction from *C. langsdorfii*, has heterogeneous monosaccharide composition (Section 2.3.1). The glucose content is low, thus; no laminaran was extracted, as it was reported previously [29]. The fraction **Cf0** was purified using anion-exchange chromatography with NaCl gradient (Section 2.3.2). **Cf1 **(1 M NaCl) and **Cf2 **(2 M NaCl) fractions were obtained. Fucoidan fraction **Cf1** had low yield and heterogeneous monosaccharide composition and thus it was put aside. The purified highly sulfated (35%) fucoidan fraction **Cf2 **contained 14% of other monosaccharides: galactose (7.9), xylose (3.0), and uronic acids (4.1). It must be noted that high sulfate content also had a galactofucan from *Sargassum mcclurei* that was recently examined by mass spectrometry [7]. The IR spectrum (not shown) of **Cf2** contained an intense absorption band at 1254 cm^−1^ (S = O) common to all the sulfate esters. An additional sulfate absorption band at 826 cm^−1^ (C–O–S, secondary equatorial sulfate) and a shoulder at 846 cm^−1^ (C–O–S, secondary axial sulfate) indicated that sulfate groups occupied positions 2 and/or 3 and position 4 of fucopyranose residues.

### 3.2. Mass Spectrometric Investigation of Fucoidan Fraction **Cf2**

Fucoidan fraction **Cf2 **with simple monosaccharide composition and high sulfate content was subjected to autohydrolysis of two different conditions to obtain LMW fragments, suitable for mass spectrometric investigation (Section 2.3.3).

The elucidation of the structural features of sulfated oligosaccharides by a negative-ion tandem mass spectrometry was carried out using the following observations, estimated in the former studies of glycosaminoglycan- [30], sulfated galactan- [16, 31, 32], and sulfated fucan-derived fragments [33, 34]. It was shown that the product ion spectra of [M-Na]^−^ (where M represents the sodium salt of oligosaccharides) featured an extensive series of B- and C-type (according to nomenclature, suggested by Domon and Costello [35]) glycosidic cleavages, whereas the Y-type cleavage occurred mainly at the sulfated residues [16, 30]. Fragment ions from cross-ring cleavage of ^0,2^X/^0,2^A-type were observed only for mono/oligosaccharides that were not 3 linked [12, 16, 30, 32, 34, 36]. As recently shown by ESIMS/MS and computational chemistry methods on C-2 and C-4-sulfated fucose residues [34], the mechanism of formation of the ^0,2^X/^0,2^A fragment ions required unsubstituted proton on the C-3 hydroxyl group. Hence, in case of 3-linked sulfated fucans/galactofucans from Laminariales [19, 20, 37] and enzymatically obtained oligosaccharides from carrageenan [14], when all protons of the C-3 hydroxyl groups are substituted, no ^0,2^X/^0,2^A-type ions could be produced during CID MS/MS.

#### 3.2.1. Mass Spectrometry of **Cf2-AH** Sample from “Soft” Autohydrolysis

**Figure 1:**
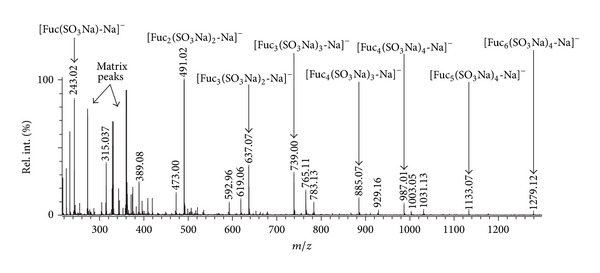
Negative-ion MALDI-TOFMS of **Cf2-AH** sample: product of “soft” autohydrolysis of **Cf2** fucoidan fraction from the brown alga *C. langsdorfii*.

The obtained preparation **Cf2-AH, **which was made in “soft” conditions (to avoid unwanted sulfate rearrangement effects [38]), due to negative-ion MALDI-TOFMS, contained a set of multiply sulfated (up to 4) oligosaccharides (DP 2–6), including monosulfated fucose residue (Figure 1). The main component of the mixture was doubly sulfated fucobiose, similarly as it was observed for the fucooligosaccharides that were derived from fucansulfate of the brown alga *F. evanescens*. As the structure of the backbone of fucansulfate from *F. evanescens* was shown [13] to have a linear core of repeating → 4)-*α*-L-Fuc*p*-(1 → 3)-*α*-L-Fuc*p*-(1 → block, it was found that during autohydrolysis 3-linked *α*-L-Fuc*p* residues hydrolyzed faster and thus oligosaccharide mixture contained monosaccharide and fragments with even DP only [1]. No sulfation at C-3 was observed for *F. evanescens* samples [1, 12, 13]. In our case, the origin of fragments with odd DP that were detected at *m/z* 637.01, 739.00, 929.16, 1031.13, and 1133.07 (Figure 1), as it was observed in autohydrolysis mixture of fucoidan from *Silvetia babingtonii* (Fucales) is unclear. The fucooligosaccharides from *S. babingtonii*, possibly, contained *α*-L-Fuc*p *residues, sulfated at position C-3 and/or insertions of 3-linked chains with DP = 2 [39], and were not yet examined by other method is but mass spectrometry. It is worthy to mention that **Cf2-AH **sample did not contain any signals corresponding to “mixed” oligosaccharides, built up of fucose and other sugars that were detected in monosaccharide composition, as it was observed in the samples from Fucales *F. evanescens* and *S. babingtonii *[1, 39], as well as *Sargassum mcclrei *[7], and Laminariales *C. costata* and* S. gurjanovae* [6, 20]. The selected conditions were assumed to be too soft for the cleavage of “mixed” oligosaccharides.

However the “softness” of the selected conditions is not soft enough to keep all the sulfates. The fact of the excess cleavage of sulfates from highly sulfated (>30%) fucans/galactofucans during autohydrolysis (and/or in the ion source of the instrument) which have a backbone with 3 type of linkage (*α*-L-Fuc*p *chain)is known [7, 19, 20].

A tandem MALDI-TOFMS of the ion at *m/z *243.02 (not shown) did not exhibit any substantial difference of that observed for *F. evanescens* sample, except that ^0,3^X-signal at *m/z* 168.9 was detected [34], like in the sample from *S. babingtonii *[39]. Minor sulfation at C-3 was also observed for fucoidans of the order Fucales: *Fucus serratus *[40] and *Fucus distichus *[41]. Furthermore, a signal that indicated sulfation at C-4 at *m/z* 182.9 (^0,2^A) had higher intensity than the signal, corresponding to sulfation at C-2 at 138.9 (^0,2^X). The observation of the prevalence of sulfation at C-4 was observed recently for the galactofucan from brown alga *Sargassum polycystum *[42].

**Figure 2:**
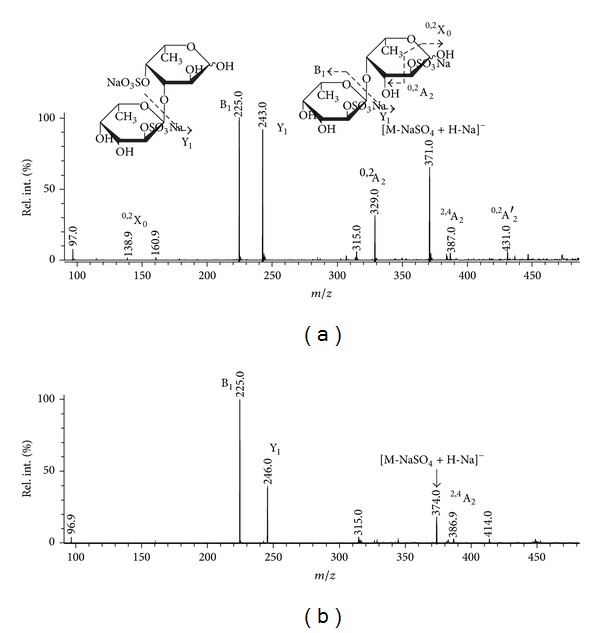
Negative-ion tandem MALDI-TOFMS ion at *m/z* 491.02. M represents sodium salt of oligosaccharide.

A tandem MALDI-TOFMS of the ion at *m/z* 491.02 (Figure 2(a)) and its reduced variant (reduction with NaBD_4_, Section 2.3.4, Figure 2(b)) had differences in comparison to that observed for *F. evanescens* sample. Fragment ions from cross-ring cleavages ^0,2^A_2_ at *m/z* 329 and ^0,2^A′_2_ at *m/z* 431, suggesting structural variants *α*-L-Fuc*p*-(2 or 4-OSO_3_^−^)-(1 → 4)-*α*-L-Fuc*p*-2-OSO_3_^−^ and *α*-L-Fuc*p-*(1 → 4)-*α*-L-Fuc*p*-2,4-di-OSO_3_^−^, had the lowest intensities along the samples from *S. babingtonii* and *F. evanescens*. A fragment ion of at *m/z *386.9 (low intensity, again) that was extected to appear as intensive ^0,2^X′-type ion [1, 39] exhibited no shift of its *m/z* to 389.9 and thus it was assumed to be ^2,4^A_2_-type fragment ion. It must be noted that the abovementioned ion was not detected in MALDI-TOF/TOFMS of the same fragment, obtained from 3-linked 2,4-disulfated *α*-L-fucan from *S. cichorioides*, obtained by autohydrolysis in the same conditions. But, its singly sulfated variant was detected at *m/z* 285 [19] also with low intensity, being probably ^0,2^X-type fragment. Fuc*p*-2-OSO_3_^−^-(1 → 3)-*α*-L-Fuc*p*-4-OSO_3_^−^.

Possibly, a high intensity of a fragment ion at *m/z *386.9 is connected with a high intensity of A-type ions, which are indicators of 4-type of linkage between fucose residues [33]. It must be noted that no A-type (except for *m/z* 386.9 and unknown ion at *m/z* 315.0) was observed at MS/MS of the reduced (Figure 2(b)) variant of the ion under study. Hence, the availability of the proton at glycosidic hydroxyl is essential for the production of the abovementioned ions of A-type. Strong signal of (B_1_-type ion) at *m/z* 225.0 from the cleavage of sulfated at C-2 *α*-L-Fuc*p* residues [31] (with loss of water molecule) along with intensive Y_1_-type ion at *m/z* 225.0 together with signal from the loss of NaSO_4_^−^ at *m/z* and small ^0,2^X-type signal at *m/z* 138.9 (indicator of 4-linked and sulfated at C-2 Fuc*p* residue on the reducing end) suggested that sulfate occupied mostly position C-4 of the reducing 3-linked Fuc*p* residue. Unfortunately, the reduction with NaBD_4_ is a destructive reaction and only major components of the mixture were successfully sequenced.

A tandem MALDI-TOFMS (not shown) of the triply sulfated fucobiose ion of low intensity at *m/z* 592.96 (not shown) was exactly the same as it was observed for the sample from *F. evanescens* [1], suggesting the following structural variants: *α*-L-Fuc*p*-2,4-di-OSO_3_^−^-(1 → 4)-*α*-L-Fuc*p*-2-OSO_3_^−^ and *α*-L-Fuc*p*-4-OSO_3_^−^-(1 → 3)-*α*-L-Fuc*p*-2,4-di-OSO_3_^−^. The selected ion had significantly higher intensity in case of *F. evanescens*.

**Figure 3:**
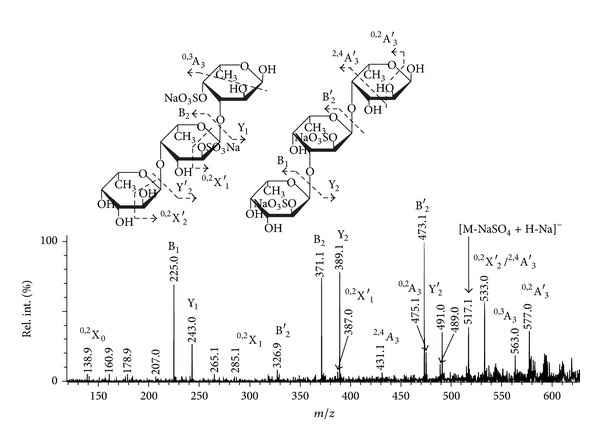
Negative-ion tandem MALDI-TOFMS ion at *m/z* 637.07. M represents sodium salt of oligosaccharide.

A tandem MALDI-TOFMS (Figure 3) of the doubly sulfated fucotriose ion at *m/z* 637.07 was very similar to the one observed for the sample from *S. babingtonii* [39] with minor differences, concerning, probably, the position of the sulfate group at the reducing *α*-L-Fuc*p* residue, since fragment ion ^0,2^X_0_ at *m/z* 138.9 was almost invisible. Thus, the following structural variants were suggested: *α*-L-Fuc*p*-2-OSO_3_^−^-(1 → 3)-*α*-L-Fuc*p*-2-OSO_3_^−^-(1 → 4)-*α*-L-Fuc*p* and *α*-L-Fuc*p*-(1 → 4)-*α*-L-Fuc*p*-2-OSO_3_^−^-(1 → 3)-*α*-L-Fuc*p*-4-OSO_3_^−^. Also, an ion of B-type (was not observed in MS/MS of the sample from *S. babingtonii*), corresponding to the cleavage of doubly sulfated fucobiose (with the elimination of water molecule) from nonreducing terminus, was found at *m/z* 327.1.

Tandem MALDI-TOF (not shown) mass spectra of the ions at *m/z* 783.13 and 885.07 (doubly and triply sulfated fucotetraoses) were very similar to that observed for the sample from *F. evanescens* [1], suggesting the following structural variants: [*α*-L-Fuc*p*-(1 → 4)-*α*-L-Fuc*p*-2-OSO_3_^−^-(1 → ]_2_, *α*-L-Fuc*p*-2-OSO_3_^−^-(1 → 4)-*α*-L-Fuc*p*-2-OSO_3_^−^-(1 → 3)-*α*-L-Fuc*p*-(1 → 4)-*α*-L-Fuc*p*, and *α*-L-Fuc*p*-2-OSO_3_^−^-(1 → 4)-*α*-L-Fuc*p*-2-OSO_3_^−^-(1 → 3)-*α*-L-Fuc*p*-2-OSO_3_^−^-(1 → 4)-*α*-L-Fuc*p*, *α*-L-Fuc*p*-(1 → 4)-*α*-L-Fuc*p*-2-OSO_3_^−^-(1 → 3)-*α*-L-Fuc*p*-2-OSO_3_^−^-(1 → 4)-*α*-L-Fuc*p*-2-OSO_3_^−^.

#### 3.2.2. Mass Spectrometry of **Cf2-exAH** Sample Obtained by “Exhaustive” Autohydrolysis

**Figure 4:**
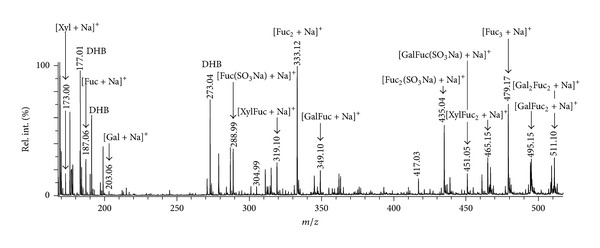
Positive-ion MALDI-TOFMS of **Cf2-exAH **sample: product of “exhaustive” autohydrolysis of **Cf2** fucoidan from the brown alga *C. langsdorfii*.

The preparation, **Cf2-exAH**, was made in “rugged” conditions. We expected to obtain a preparation with a higher degree of hydrolysis to observe minor constituents. A mixture **Cf2-exAH** contained both desulfated and sulfated LMW fragments, visible both in positive- (Figure 4) and negative-ion mode MALDI-TOFMS (not shown). Ions of “mixed” oligosaccharides, built up of fucose and galactose residues, were clearly observed in a positive ion mode at *m/z* 349.10, 495.15, and 511.10. However, informative spectra were recorded only for positive-ion mode. Negative-ion tandem mass spetrum of [GalFucSO_3_]^−^ ion at *m/z* 405.02 (not shown) exhibited only B-type ions at *m/z* 241.0 and 225.0 from the cleavage of sulfated at C-2 Gal and Fuc residues, respectively. Xylose-containing fragments (*m/z* 173.00 and 319.10, Figure 4) were not detected in negative-ion mode; thus, xylose residues were assumed to be free of sulfates. Some interpretable tandem spectra that were recorded with minimal interference from closer ions of other compounds are depicted below.

**Figure 5:**
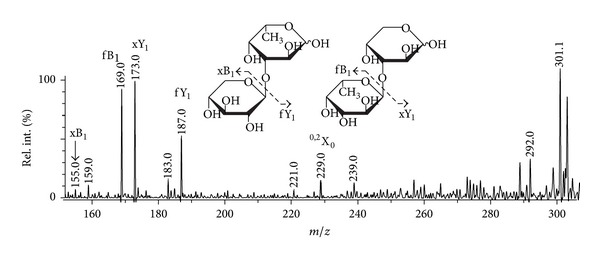
Positive-ion tandem MALDI-TOFMS ion at *m/z* 319.10.

A positive-ion tandem MALDI-TOFMS (Figure 5) of the “mixed” ion at *m/z* 319.10 exhibited main signals from the cleavages of glycosidic bonds. The only information we could extract is that xylose residues could occupy both reducing and nonreducing positions. The presence of an ion at *m/z* 229.0 informs that mixture contains species different from (1 → 3)-type of linkages.

**Figure 6:**
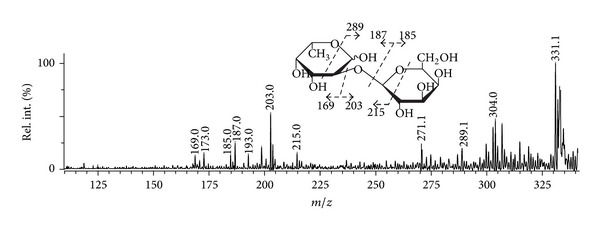
Positive-ion tandem MALDI-TOFMS ion at *m/z* 349.10.

A positive-ion tandem MALDI-TOFMS (Figure 6) of the ion at *m/z* 349.10 of the disaccharide, built up of fucose and galactose, was more interesting. The fragmentation pattern indicated that galactose (by unique fragment at *m/z* 215), probably, resided as a 2-linked branching point from the main chain of fucoidan that was observed in the recent work of Bilan et al. 2013 on the structural features of the galactofucan from *S. polycystum* [42].

**Figure 7:**
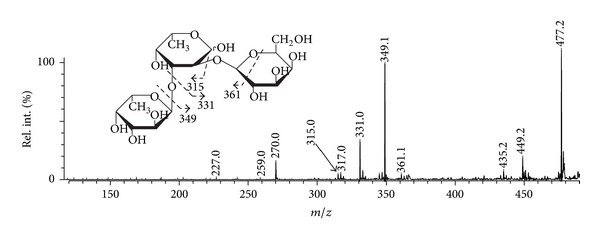
Positive-ion tandem MALDI-TOFMS ion at *m/z* 495.15.

Again, a positive-ion tandem MALDI-TOFMS (Figure 7) of the ion at *m/z *495.15 of a trisaccharide, built up of 2 fucose and 1 galactose residues demonstrated unique signal at *m/z* 361.1, probably, from 1,5-type of cross-ring cleavage of hexose ring, that was not observed in xylose-containing oligosaccharides. The lack of a characteristic ion at *m/z* [M-60], where M represents sodium adduct of oligosaccharide, indicated that fucose residues were 3-linked.

## 4. Conclusions

Two fractions of fucoidan were extracted from brown alga *Coccophora langsdorfii*, which was collected in July 2008. The highly sulfated (35%) fucoidan fraction **Cf2 **with simple monosaccharide composition, which contained, along with fucose, galactose and traces of xylose and uronic acids was characterized. Its structural features were predominantly determined (in comparison with fragments of known structure) by a rapid mass spectrometric investigation of the low-molecular-weight (LMW) fragments, obtained by “mild” and “exhaustive” autohydrolysis. Tandem MALDI-TOFMS of fucooligosaccharides with even DP (degree of polymerization) were exactly the same as that observed for fucoidan from *F. evanescens*, which have a backbone of alternating sulfated (1 → 3)- and (1 → 4) linked sulfated *α*-L-Fuc*p* residues [13]. Fragmentation patterns of oligosaccharides with odd DP indicated sulfation at C-2 of 4-linked *α*-L-Fuc*p* residues and at C-4 of (1 → 3) linked *α*-L-Fuc*p* residues on the reducing terminus. Minor sulfation at C-3 was detected by investigation of monosulfated fucose, and also a prevalence of the sulfation at C-4 over sulfation at C-2 was observed. The “exhaustive” autohydrolysis (maximal concentration in minimal volume) allowed us to obtain a mixture, enriched with minor constituents of the **Cf2** fucoidan—xylose and galactose. It was found that unsulfated xylose residues could be, probably, 3- or 4-linked to fucose residues and could reside both on the reducing and nonreducing termini. Non-reducing galactose residues were found to be linked (in GalFuc disaccharides), possibly, by 2-type of linkages to fucose residues that were sulfated, most likely, at position C-2. Structural features of trisaccharides Gal_2_Fuc were not established due to strong overlapping of peaks. Fragments, including uronic acids, were not detected, being probably, minor impurities.
